# Sex Modifies the Effect of COVID-19 on Arterial Elasticity

**DOI:** 10.3390/v16071089

**Published:** 2024-07-06

**Authors:** Jared C. Durieux, Sokratis N. Zisis, Christian Mouchati, Danielle Labbato, Marc Abboud, Grace A. McComsey

**Affiliations:** 1 University Hospitals Cleveland Medical Center, Cleveland, OH 44106, USA; jared.durieux@uhhospitals.org (J.C.D.); danielle.labbato@uhhospitals.org (D.L.); 2 School of Medicine, Case Western Reserve University, Cleveland, OH 44106, USA; sokratis.zisis@case.edu (S.N.Z.); cfm71@case.edu (C.M.); 3 Faculty of Medicine, Saint Joseph University of Beirut, Beirut 1104 2020, Lebanon; marc.abboud@net.usj.edu.lb

**Keywords:** COVID-19, long COVID, PASC, endothelial dysfunction, arterial stiffness, effect modification, sex differences

## Abstract

There is limited long-term evidence on the effects of COVID-19 on vascular injury between male and female sex. An adult cohort of COVID-19 survivors (COVID+) and confirmed SARS-CoV-2 antibody-negative participants (COVID-) were prospectively enrolled. COVID+ participants who have documented the presence of persistent symptoms four weeks following infection were considered to have post-acute sequelae of COVID-19 (PASC). Non-invasive, FDA-approved EndoPAT (Endo-PAT2000) was used for endothelial assessment. COVID-(*n* = 94) were 1:1 propensity score matched to COVID+ (*n* = 151) on baseline covariates including sex. Among COVID+, 66.2% (*n* = 100) had PASC. Higher levels of coagulation marker, D-dimer (*p* = 0.001), and gut permeability marker, zonulin (*p* = 0.001), were associated with female sex. Estimated differences in augmentation index (AI) between COVID− (0.9 ± 17.2) and COVID+ (8.4 ± 15.7; *p* = 0.001) and between female and male sex (12.9 ± 1.9; *p* < .0001) were observed. Among COVID+ with PASC, the average AI (10.5 ± 1.6) was 9.7 units higher than COVID− (*p* < .0001) and 6.2 units higher compared to COVID+ with no PASC (*p* = 0.03). COVID+ PASC+ female sex had the highest AI (14.3 ± 1.9). The effects of SARS-CoV-2 infection on vascular function varies across strata of sex and female sex in the post-acute phase of COVID-19 have the worse arterial elasticity (highest AI).

## 1. Introduction

Sex differences in COVID-19 disease susceptibility and severity have been observed, notwithstanding similar infection rates between male and female sex [1]. Male sex have a higher risk of SARS-CoV-2 infection, is a risk factor for acute COVID disease severity, and have 1.7 times higher risk of death compared to female sex [2,3,4]. Long-term outcomes have been observed more frequently in female sex, independent of acute COVID severity, and female sex are more likely to suffer from ongoing fatigue, anxiety, and depression [5,6]. Despite evidence of biological differences in response to COVID morbidity and mortality between male and female sex, sex as a potential effect modifier is rarely discussed.

COVID-19 is characterized by hyperinflammation and dysregulated immune response and has been associated with poor cardiovascular outcomes [7,8]. The high levels of cytokines and increased angiotensin-converting enzyme 2 (ACE-2) expression observed among acute COVID-infected survivors are known to affect cardiovascular systems [9,10]. Damage to the vascular endothelium, which is prolifically evident in SARS-CoV-2 infections, [11] is known to increase the risk of incident cardiovascular disease (CVD) although the rate of vascular injury depends on sex [12,13]. CVD is the number one cause of death for men and women, yet women experience two times higher burden of heart failure and coronary microvascular dysfunction [14].

Risk factors such as older age, hypertension, diabetes, obesity, and smoking that are associated with COVID-19 disease are known to affect arterial distensibility. The overlapping risk factors between COVID-19 disease and endothelial dysfunction may have a biological basis and a shared phenotype. This association suggests that the underlying mechanisms are heterogenous. The purpose of our study was to assess the effect of COVID-19 on endothelial function and arterial stiffness and determine whether those differences vary across strata of sex. Understanding the effects of SARS-CoV-2 infection between male and female sex on vascular function will provide the opportunity to identify high risk groups and develop sex-specific therapies.

## 2. Materials and Methods

An adult cohort (age ≥ 18 years) was prospectively enrolled at University Hospitals Cleveland Medical Center, Cleveland, Ohio, between January 2020 and January 2021. Participants were either SARS-CoV-2 antibody-negative with no prior history of COVID-19 infection, suggestive symptoms, or acute respiratory illness since December 2019 (COVID-) or COVID-19 survivors with a documented history of COVID-infection regardless of severity of the initial illness (COVID+). Among COVID+, participants with symptoms that persisted four weeks or more following the infection were considered to have post-acute sequelae of COVID-19 (COVID+ PASC+). Although viral genomes of COVID-infected study participants were not sequenced to identify which variant was prevalent, the predominant variants during our study period included the wildtype strain (early 2020), D614G variant (mid-to-late 2020), and the alpha variant (B.1.1.7) from late 2020 to early 2021.

Following consent, a detailed review of participant’s medical history was obtained by a trained clinician that included co-morbidities, clinical diagnoses, and COVID-19 infection date. Body mass index (BMI) was derived from measured height and weight and fasting (12 h fast) blood samples were obtained for measurements of lipids and biomarkers using enzyme-linked immunosorbent assay (ELISA). Biomarkers included the inflammation marker, high-sensitivity C-reactive protein (hs-CRP) and oxidized low-density lipoprotein (oxLDL) using the relevant kits (Upsala, Mercodia, Sweden), the coagulation marker D-dimer (Diagnostica Stago, Parsippany, NJ, USA), and a known marker of gut permeability, zonulin-1 (Promocell, Germany).

Non-invasive, FDA-approved EndoPAT (Endo-PAT2000) was used for endothelial assessment by post-occlusive reactive hyperemia arterial tonometry (RH-PAT). Arterial elasticity was measured by Augmentation Index (higher AI = worse arterial stiffness) and standardized to 75 heart beats per minute (AI@75). Endothelial function was measured by Reactive Hyperemia Index (RHI) where RHI ≥ 1.67 was considered normal. Prior to endothelial assessment, participants were asked to fast from food and vitamin intake (including caffeine), aerobic activity, and tobacco use 12 h prior to assessment with no use of vasodilator medications 4 h prior to assessment. Additional details on EndoPat assessment are described in Appendix A.

### Statistical Methods

A 1:1 propensity score matched (PSM) sample from the observed data of uninfected controls were matched to cases of confirmed COVID-survivors on observed baseline covariates that included age, sex, race, BMI, smoking status, hypertension, and diabetes using matching with replacement. Each COVID+ participant’s propensity score was randomly matched to the same sex among COVID− if the difference in the logits of the propensity score were ≤0.2. Covariate balance and bias reduction was assessed using the standardized difference ((100*(μ(COVID) − (μ(controls))))⁄√(((〖SD〗^2 (COVID)+〖SD〗^2 (controls))/2)). Baseline characteristics of study participants were summarized as mean ± standard deviation (SD), median and interquartile range (IQR), or frequency (n) and percentage (%). Differences between groups were computed using Wilcoxon rank sum for continuous measures and Chi-squared or Fisher’s test for categorical variables.

Logistic regression was used to assess risk factors associated with female sex and generalized linear mixed models were used to estimate the stratum-specific effect of COVID on endothelial function (RHI) and arterial elasticity (AI) across strata of sex. Adjusted models included age, sex, race, systolic blood pressure, lipids, body mass index (BMI), smoking status, and having either pre-existing hypertension or diabetes. All analyses were conducted using SAS 9.4 (SAS Inc., Cary, NC, USA) and *p*-values less than alpha < 0.05 were considered statistically significant.

## 3. Results

### 3.1. Characteristics

In our sample (Table 1), 151 (*n* = 61.6%) participants were COVID+ and 94 (38.4%) were COVID−. The average age among COVID+ was 5 years older compared to COVID−(45.9 vs. 40.9 years; *p* = 0.01) and both groups had similar BMIs (29 kg/m^2^; *p* = 0.6). The proportion of female sex, non-white race, and participants with hypertension or diabetes at baseline, taking angiotensin converting enzyme inhibitors or angiotensin receptor blockers, or statins were similar (*p* > 0.05) between the groups. Among COVID-19 survivors, the median number of days since COVID diagnosis was 249 (IQR: 139.0, 510.0) and 66.2% (*n* = 100) had PASC. More than half (*n* = 53) of COVID+ PASC+ experienced at least seven persistent symptoms attributable to long COVID. Fatigue (being very tired; *n* = 65), problems thinking or concentrating (brain fog; *n* = 64), pain in any part of your body (*n* = 54), problems with anxiety, depression, stress, or trauma-related symptoms like nightmares or grief (*n* = 52), and post-exertional malaise (symptoms worse after even minor physical or mental effort; *n* = 49) were the most commonly reported symptoms.

#### 3.1.1. Associations with Female Sex

As outlined in Table 2, for every one-unit increase in AI was 81% [uOR: 1.8 (95% CI: 1.3, 2.6); *p* = 0.002)] more likely associated with female sex compared to male sex. Every unit increase in D-dimer was 57% [uOR: 1.6 (95% CI: 1.1, 2.2); *p* = 0.001] more likely associated with female sex compared to male sex and every unit increase in zonulin was nearly two times [uOR: 1.9 (95% CI: 1.3, 2.7); *p* = 0.001] more likely to be associated with female sex compared to male sex. After adjusting for COVID status, age, race, BMI, non-HDL cholesterol, systolic blood pressure, smoking status, and baseline comorbidities (hypertension and diabetes), the effects of AI, BMI, D-dimer, and zonulin remained independently associated (*p* < 0.05) with female sex. There was insufficient evidence to suggest that RHI, age, race, current smoker, preexisting hypertension or diabetes, IL-6, or hs-CRP were associated with sex.

#### 3.1.2. Endothelial Assessment by COVID-19 Status and Sex

**Figure 1:**
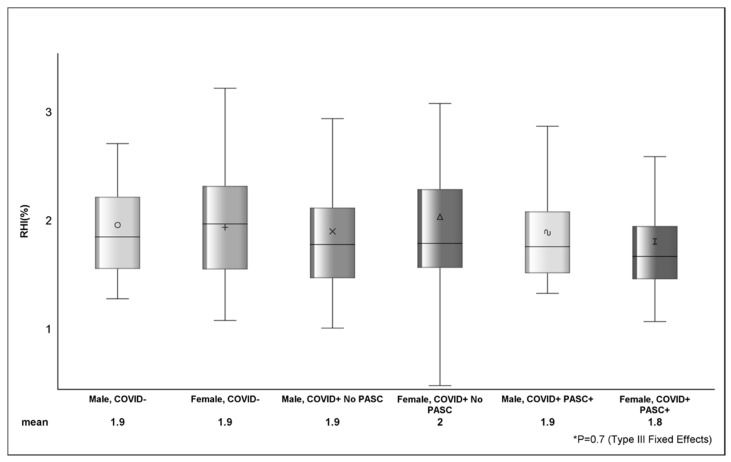
Figure shows the distribution of reactive hyperemia index between sex, COVID, and PASC status. The various shapes within the box represent the mean (value depicted at bottom of figure) and the horizontal bar is the median. The lower (Q1 or 25th percentile) and upper (Q3 or 75th percentile) parts of the box make up the interquartile range (IQR) and the error bars represent the minimum and maximum observed values. *p*-value (*p* = 0.7) from Type III Fixed Effects is testing the difference in means between groups from linear mixed-effects model.

Among COVID−, the average RHI was 1.89%, and 42.6% (*n* = 40) had RHI ≤ 1.67 (Table 3). The mean RHI among COVID+ was 1.87%, and 43.7% (*n* = 66) had RHI ≤ 1.67. The estimated difference in AI between COVID− (0.9 ± 17.2) and COVID+ (8.4 ± 15.7) was 7.5 (*p* = 0.001) and between female and male sex was 12.9 ± 1.9 (*p* <.0001). There were no observed differences (*p* > 0.05) in RHI (Figure 1) or RHI ≤ 1.67 between COVID status or across strata of sex.

#### 3.1.3. Associations with Arterial Elasticity as Outcome

**Figure 2:**
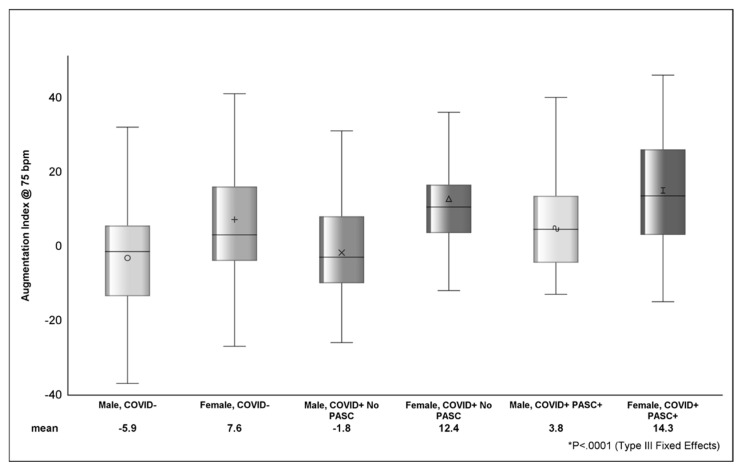
Figure shows the distribution of AI between sex, COVID, and PASC status. The various shapes within the box represent the mean (value depicted at bottom of figure) and the horizontal bar is the median. The lower (Q1 or 25th percentile) and upper (Q3 or 75th percentile) parts of the box make up the interquartile range (IQR) and the error bars represent the minimum and maximum observed values. *p*-value (<.0001) from Type III Fixed Effects is testing the difference in means between groups from linear mixed-effects model.

Looking at the distribution of AI across strata of sex (Figure 2), COVID+ PASC+ female sex had the highest AI (14.3 ± 1.9), followed by COVID+ with no PASC female sex (12.4 ± 3.2) and COVID− female sex (7.7 ± 2.2). The difference in AI between COVID+ PASC+ male sex (3.8 ± 2.5) and COVID+ PASC+ female sex was 10.5 (*p* <.001). In Table 4, the estimated AI was highest among COVID+ PASC+ (9.1 ± 2) and lowest among COVID-participants (−1.2 ± 1.6). Compared to male sex, female sex was associated with higher AI (9.3 ± 2.2; *p* <.0001). D-dimer (*p* = 0.0002) and zonulin (*p* = 0.01) were positively associated with AI; however, the effects of these markers on AI was attenuated (*p* > 0.05) in adjusted models.

## 4. Discussion

In our study, we demonstrated that the effects of SARS-CoV-2 infection on augmentation index vary across strata of sex and that female sex in the post-acute phase of COVID-19 have the worse arterial elasticity (highest AI). Additionally, we provided evidence that SARS-CoV-2 infection, PASC, and sex were independently associated with AI and that higher levels of AI, as well as coagulation marker D-dimer, and gut permeability marker zonulin were associated with female sex. This extends our previous findings that showed the association of arterial elasticity, inflammation [15], and gut permeability [16] with PASC.

Effect modification occurs when the effect of one variable on another differs across strata of a third variable [17] with the goal of identifying sub-groups to target for treatment. In our sample, both the proportion of male and female sex within and between COVID groups were similar and sex, as well as COVID and PASC status, was found to be independently associated with AI. The overall crude estimate of AI—an average of the stratum-specific estimates among COVID+ (our exposure)—was nearly 39% lower than the stratum-specific estimate for COVID-infected female sex. Without stratification by sex, the effect of COVID on AI would either be underestimated for female sex or overestimated for male sex.

Since COVID-infection, PASC, and sex were independently associated with AI and—as we already know—the effect of COVID-infection on AI is neither the result of sex nor depends on sex, then this suggests that the effect of COVID on AI is not a result of an interaction between COVID and sex. Overall, the stratified-specific estimates of AI by sex, combined with the evidence that the effect of COVID-infection on AI does not depend on sex, suggests that sex is an effect modifier of COVID-infection on arterial elasticity. To our knowledge, this is the first study to establish that sex is an effect modifier of SARS-CoV-2 infection on arterial elasticity among COVID-survivors with PASC.

The mechanisms underlying the sex-specific disparity in PASC are not fully understood, but interactions between sex hormones and the gut microbiome may contribute. It is known that there is a mutual interaction between sex steroids and the gut microbiota, interactions that play a prominent role in the development of metabolic diseases [18]. Estrogens produced in the body can be metabolized by gut microbes, with the resultant metabolites influencing the host [19]. Additionally, sex hormones directly modulate the metabolism of bacteria through steroid receptors [20]. The observed sex differences in gut microbiota composition have led to multiple studies highlighting the interaction between steroid hormones and the gut microbiota, as well as its influence on diseases like obesity and diabetes [21]. Our finding of sex-related differences in the gut permeability marker zonulin among COVID-survivors with PASC is novel and deserves further investigation.

Another possible explanation of our findings is the difference in immune system function between male and female sex. It has been observed that female sex have more robust T-cell activation and higher levels of cytokines during infections [22]. Female sex exhibit more rapid and robust innate and adaptive immune responses, which can aid in initial infection protection and severity mitigation [23] whereas viral RNA clearance may be delayed in male sex [24]. This could explain the sex differences observed in long COVID syndrome [25]. Female sex typically exhibit more rapid and robust innate and adaptive immune responses, which can aid in initial infection protection and severity mitigation. However, this same immune system difference can make female sex more susceptible to prolonged autoimmune-related diseases [26]. The hypothesized theory that SARS-CoV-2 fragments could remain hidden in reservoirs throughout the body, igniting chronic inflammation-associated cascades that give rise to long COVID symptoms may explain the worse arterial stiffness noted in our study [27]. Additionally, Stewart et al. suggested that given the overlapping symptoms between long COVID syndrome and perimenopause and menopause, sex hormone differences may play a role in the asymmetry of risk and outcomes between male and female sex [28]. Prior coronavirus outbreaks have also reported sex differences in outcomes, indicating the importance of considering sex-specific clinical insights when treating COVID-19 patients [29,30,31].

Previous studies have largely explored the effects of COVID-19 on various markers of vascular function. One study showed that there was significantly lower vascular function and higher arterial stiffness among young adults occurring several weeks after contracting COVID-19 compared to healthy controls [32]. However, this study does not account for vascular changes that may occur over time. In another study evaluating the effects of COVID-19 6 months following the infection in a cohort of young adults demonstrated that levels of carotid femoral pulse wave velocities were significantly reduced, while arterial stiffness remained unaltered over time [33]. While this study observed changes over a short time period there was lack of baseline data to accurately attribute these changes to COVID-infection. One retrospective, multicenter cohort study demonstrated that arterial stiffness in hospitalized patients for COVID-19 was associated with higher all-cause mortality [34]. One case-control study that utilized a comparison of brachial–ankle and carotid–femoral pulse wave velocities determined that arterial stiffness was higher in patients with COVID-19 compared to those without COVID-19 [35], suggestive of the acute effects of COVID-infection on endothelial function.

There are limited studies that specifically investigate the association between sex and arterial elasticity in post-acute sequalae of SARS-CoV-2. One previous study reported that older age, sex, and time from onset of the infection may be important determinants in influencing arterial stiffness among a cohort of patients with long-COVID [36]. Another recent study demonstrated that increased arterial stiffness, reduced aortic elasticity, and diastolic dysfunction were common among a cohort of female sex with PASC and were associated with the time from onset of diagnoses [37]. However, these studies either lacked sufficient evidence to identify sex differences in endothelial function, were missing appropriate comparison groups, included participants with prior cardiovascular complications or metabolic syndrome, or did not report enough information to determine if the included sample met the formal criteria of experiencing PASC.

By measuring arterial stiffness, the augmentation index reflects the risk of cardiovascular disease [38]. Aortic stiffness has been associated with a decreased quality of life [39] and mental illness including depression [40]. Therefore, measuring AI in the post-acute phase of COVID-19 will help assess post-COVID severity and monitor its progression to help establish a treatment plan. One of the proposed measures to improve the AI is cardiopulmonary rehabilitation, which improves patients’ functional status and exercise capacity, thus enhancing the quality of life and preventing long-term complications [41,42]. Pulmonary rehabilitation has also been shown in one study to improve endothelial function in convalescent COVID-19 patients [43]. 

## 5. Conclusions

Future studies should assess arterial elasticity in the post-acute phase of COVID-19 to further shed light on the long-term effects of COVID. Immediate interventions should focus on female sex as a high-risk group. Additional studies are needed to better understand the etiology of PASC and develop effective sex-specific therapeutic strategies and public health interventions.

## Figures and Tables

**Table 1:** Characteristics of Participants by COVID-19 Status *.

				COVID+ (*n* = 151)		COVID− (*n* = 94)	*p*-Value
				n (%) or Median (IQR)/Mean ± Std			
Demographics							
	Age (years)			45.9 ± 13.4		40.9 ± 14.2	0.01
	Sex (Female)			86 (56.9)		47 (50.0)	0.3
	Non-white Race *			46 (30.5)		36 (38.3)	0.2
	BMI (kg/m^2^)			29.9 ± 6.4		29.3 ± 5.6	0.6
	Current Smoker (Yes)			26 (17.2)		28 (29.8)	0.02
	Hypertension at Baseline (Yes)			33 (21.9)		13 (13.8)	0.1
	Diabetes at Baseline (yes)			8 (5.3)		4 (4.3)	0.7
	Number of days since COVID-19 Diagnosis			249.0 (139.0, 510.0)		--	--
	Total Number of self-reported PASC Symptoms **						
		1 to 6		47 (31.1)		--	--
		≥7		53 (35.1)		--	--
Current Medications							
	ACE or ARB (Yes) ***			13 (8.6)		7 (2.9)	0.7
	Beta Blocker (Yes)			6 (3.9)		0	0.08
	Statin (Yes)			8 (5.3)		9 (9.6)	0.2
Laboratory Data							
	Cholesterol (mg/dL)			180.8 ± 33.9		176.2 ± 33.8	0.3
	non-HDL (mg/dL) ****			127.8 ± 36.2		126.5 ± 39.5	0.5
	Triglycerides (mg/dL)			119.3 ± 70.8		126.3 ± 129.9	0.7
Biomarkers							
	Coagulation; D-dimer (ng/mL)			415.6 (257.3, 617.1)		358.3 (204.0, 584.5)	0.2
	OxLDL *****			76.6 ± 32.1		53.9 ± 24.9	<.0001
	Inflammation; hs-CRP (ng/mL)			2792.9 (1193.2, 7241.8)		2595.6 (818.5, 7525.1)	0.3
	Gut permeability; Zonulin (mg/mL)			4282.9 (2462.8, 6506.5)		3053.9 (1947.9, 5165.0)	0.01

* Includes African American, Asian, Hispanic, and Other, ** COVID+ PASC+ (only), *** Angiotensin converting enzyme inhibitors or angiotensin receptor blockers, **** non-high-density lipoprotein cholesterol, ***** Oxidized Low-density lipoprotein (per 1000).

**Table 2:** Risk Factors Associated with Female Sex *.

		uOR (95% CI); *p*-Value **	aOR (95% CI); *p*-Value ***
COVID-19 Status (+ vs. −)		1.32 (0.79, 2.22); *p* = 0.3	1.2 (0.68, 2.12); *p* = 0.5
BMI (kg/m^2^)		1.05 (1.01, 1.11); *p* = 0.03	1.06 (1.01, 1.11); *p* = 0.04
non-HDL (mg/dL)		3.18 (1.29, 7.81); *p* = 0.01	2.49 (0.91, 6.83); *p* = 0.08
OX-LDL		2.01 (1.1, 3.67); *p* = 0.02	1.7 (0.85, 3.55); *p* = 0.1
Ddimer (ng/mL)		1.57 (1.1, 2.24); *p* = 0.01	1.93 (1.23, 3.05); *p* = 0.01
Zonulin (mg/mL)		1.86 (1.27, 2.73); *p* = 0.001	1.89 (1.25, 2.84); *p* = 0.002

* Table displays estimates for COVID-19 status and characteristics that were associated (*p* < 0.05) with female sex in unadjusted models. ** uOR = unadjusted odds ratio; CI = confidence interval. *** aOR = adjusted odds ratio; adjusted models included COVID-19 status, race, baseline BMI, non-HDL, systolic BP, smoking status, comorbidities (hypertension, diabetes), and oxLDL, D-dimer, and zonulin were modeled separately.

**Table 3:** Endothelial Function and Arterial Elasticity by Sex *.

			COVID+		COVID−	*p*-Value **
			Mean ± Std/n(%)			
Augmentation Index @75 bpm						
	Overall		8.44 ± 15.73		0.88 ± 17.15	0.001
		Female	13.83 ± 16.27		7.64 ± 16.38	
		Male	1.31 ± 11.73		−5.87 ± 15.27	
Reactive Hyperemia Index (%)						
	Overall		1.87 ± 0.63		1.89 ± 0.59	0.9
		Female	1.86 ± 0.56		1.87 ± 0.5	
		Male	1.88 ± 0.69		1.9 ± 0.67	
Reactive Hyperemia Index (≤1.67)						
	Overall		66 (43.7)		40 (42.6)	0.8
		Female	41 (47.67)		19 (40.43)	
		Male	25 (38.46)		21 (44.68)	

* Outcomes are measures of endothelial function, COVID-19 status is the exposure, and sex is the effect modifier. ** *p*-value is assessing the difference in each outcome between COVID+ and COVID− by sex.

**Table 4:** Associations with Augmentation Index *.

		Unadjusted	*p*-Value	Adjusted **	*p*-Value ***
		Estimate ± s.e.		Estimate ± s.e.	
COVID-19 and PASC STATUS			0.001		0.002
	COVID+ PASC+	9.06 ± 1.99		11.84 ± 2.4	
	COVID+ No PASC	0.92 ± 2.64		5.53 ± 2.72	
	COVID-	−0.17 ± 1.59		5.47 ± 2.25	
SEX (F vs. M)		9.26 ± 2.23	<0.0001	14.86 ± 1.81	<0.0001
OxLDL		0.07 ± 0.04	0.1	0.03 ± 0.04	0.4
D-dimer		5.22 ± 1.39	0.0002	−1.85 ± 1.12	0.1
Zonulin		3.52 ± 1.45	0.02	−0.8 ± 1.21	0.4

* Estimates of Augmentation Index were computed using linear mixed models. ** Adjusted models included age, race, BMI, smoking status, lipids, and pre-existing comorbidities. *** *p*-value = Type III Tests of Fixed Effects.

## Data Availability

Data are available upon request.

## Appendix A

Peripheral arterial tonometry measured using the EndoPAT is among the methods for testing endothelial function in humans and has been shown to independently predict cardiovascular events providing additional prognostic information beyond the conventional CVD risk factors [44].

Assessing endothelial function using an EndoPat device is a non-invasive, highly reproducible, and less operator-dependent method compared to the cumbersome flow-mediated dilation. EndoPAT records the plethysmographic pressure changes that are caused by the arterial pulse and then translates this information into a peripheral arterial tone. This instrument provides two indexes. The first is RHI, which indicates the peripheral endothelium-dependent vasodilator capacity (nv > 1.67). The second is AI, which measures the arterial stiffness by reporting the difference between first and second peaks of the arterial waveform (AI (%) = (P − 2P1)/P1).

The participant sits in a reclining chair with their hands at heart level and fingers hanging freely. Fingertip probes are placed on both index fingers and the pulse wave amplitudes are recorded for the duration of the procedure. There is a 5 min baseline recording followed by a 5 min occlusion of non-dominant arm using a blood pressure cuff inflated to 40 mmHg above systolic pressure. The last 5 min there is rapid deflation of BP cuff followed by reactive, flow-mediated hyperemia and pulse wave amplitudes recording. An integrated software program compares the ratio of arterial pressure waves in the two fingers before the occlusion and after the deflation to calculate the RHI score as a ratio of the average pulse wave amplitude, measured over 60s and starting one minute after cuff deflation to the average pulse wave amplitude measured at baseline. This ratio is normalized to the concurrent signal from the contralateral finger to correct for changes in the systemic vascular tone. The ratio of the difference between picks of pressure in anacrote and the pulse pressure expressed as a percentage that is standardized to 75 heart beats per minute (AI@75) is also calculated. This is a measure of arterial stiffness where lower or negative AI values indicate better arterial elasticity [45,46].
